# Paclitaxel-Induced Hypertriglyceridemia Complicated by Acute Pancreatitis: A Case Report

**DOI:** 10.7759/cureus.99411

**Published:** 2025-12-16

**Authors:** Rahul Chowdhary, Kirti Arora, Rishi Chowdhary, Sarmand Jameel, Manjeet K Goyal, Elliot Sorrell

**Affiliations:** 1 Internal Medicine, North Delhi Municipal Corporation Medical College & Hindu Rao Hospital, New Delhi, IND; 2 Medicine, Dayanand Medical College and Hospital, Ludhiana, IND; 3 Internal Medicine, SDM College of Medical Sciences and Hospital, Dharwad, IND; 4 Internal Medicine, Willis Knighton Health, Shreveport, USA; 5 Gastroenterology and Hepatology, Dayanand Medical College and Hospital, Ludhiana, IND

**Keywords:** acute pancreatitis, breast cancer, chemotherapy, hypertriglyceridemia, paclitaxel

## Abstract

Paclitaxel, a widely used chemotherapy agent, has been associated with various adverse effects, including hypertriglyceridemia and, rarely, acute pancreatitis. The potential for paclitaxel to trigger lipid metabolism disturbances necessitates close monitoring of lipid profiles in patients undergoing treatment.

We report the case of a 48-year-old woman with a history of breast cancer who developed acute pancreatitis following paclitaxel chemotherapy. She presented with abdominal pain radiating to the back, nausea, and vomiting one week after her fourth cycle of paclitaxel. Imaging confirmed acute pancreatitis, and laboratory studies revealed significantly elevated serum triglyceride and lipase levels. Paclitaxel was discontinued, and the patient was managed with lipid-lowering therapy, analgesics, and intravenous hydration, leading to clinical improvement and normalization of triglyceride and lipase levels. She was subsequently referred back to the oncology team for further guidance on her breast cancer therapy. Given her paclitaxel-based regimen, chemotherapy-induced hypertriglyceridemia complicated by pancreatitis was strongly suspected.

This case highlights the importance of early recognition and monitoring of lipid levels in patients receiving paclitaxel chemotherapy, as prompt intervention can prevent severe complications. Further research is warranted to elucidate the mechanisms and risk factors for this rare but life-threatening adverse effect.

## Introduction

Acute pancreatitis is a frequently observed gastrointestinal ailment that necessitates hospitalization in the United States. It is estimated that the occurrence of acute pancreatitis ranges from 110 to 140 cases per 100,000 people, with over 300,000 emergency department visits per year. From 2002 to 2013, admissions owing to acute pancreatitis rose from 9.48 cases per 1000 hospitalizations to 12.19, with an average hospitalization expense of almost $7000 [1]. Around 80% of patients experience a mild to moderately severe form of the disease, while one out of five individuals develops a severe illness that carries a mortality rate of approximately 20% [1].

Gallstones (30-40%) and alcohol (30-35%) are the most frequent causes of acute pancreatitis. Other important etiologies include hypertriglyceridemia, post-endoscopic retrograde cholangiopancreatography (ERCP) pancreatitis, and a growing list of medication-related causes. Drugs are a rare cause, accounting for only 0.1%-2% of all cases, and are typically diagnosed by excluding other potential causes [2]. The World Health Organization (WHO) has identified over 500 drugs that can cause acute pancreatitis as a drug-related side effect. Although most cases of drug-induced acute pancreatitis (DIAP) are mild to moderate in severity, severe and even life-threatening presentations have also been documented [2].

The most frequently reported symptom when seeking medical attention is abdominal pain. The abdominal pain associated with acute pancreatitis is typically described as continuous, and it may extend to the back, becoming more pronounced when eating, drinking, or lying flat on the back [1]. In order to identify drug-induced acute pancreatitis, the initial step involves confirming the presence of acute pancreatitis. As per the Atlanta criteria, a diagnosis of acute pancreatitis requires the presence of at least two out of three criteria: experiencing abdominal pain consistent with acute pancreatitis, elevated serum levels of amylase and/or lipase that are three times higher than the normal range, and observable abnormalities on imaging tests [3].

Paclitaxel is a significant drug in the treatment of breast cancer. It has shown efficacy in various types of breast cancer through numerous adjuvant and neoadjuvant clinical trials. As a result, it can be utilized as a first-line therapy, second-line therapy, salvage therapy, or as an adjuvant or neoadjuvant treatment. Additionally, it can be used in combination with other agents to enhance its therapeutic effects [4]. Despite its benefits, paclitaxel is associated with toxicities and side effects like hypersensitive reaction, cardiac toxicities, neutropenia, peripheral neuropathy, and chemoresistance [4]. Although not routinely recognized as a lipid-altering agent, paclitaxel has been associated in rare reports with secondary hypertriglyceridemia, which can, in turn, precipitate acute pancreatitis.

Hypertriglyceridemia is a type of dyslipidemia characterized by abnormally high levels of triglycerides in the bloodstream [5]. While dyslipidemia is not currently recognized as a confirmed side effect of these chemotherapeutic agents, elevated triglyceride levels can have significant consequences, such as an elevated risk of cardiovascular disease and acute pancreatitis [6].

Here, we report a rare case of paclitaxel-induced severe hypertriglyceridemia complicated by acute pancreatitis in a patient previously undiagnosed with hyperlipidemia and the corresponding therapeutic intervention. Through this case, we want to highlight the importance of monitoring triglyceride levels during paclitaxel therapy to raise awareness of this uncommon but clinically significant complication.

## Case presentation

We encountered a 48-year-old female with a past medical history of chronic hypertension (duration not specified in the available records) and breast cancer (T3N1M0, status post [s/p] right radical mastectomy with lymph node biopsy and total left mastectomy), diagnosed in June 2022. She had a height of 1.63 m, a weight of 80.5 kg, a body surface area (BSA) of 1.91 m², and a body mass index (BMI) of 30.5 kg/m². The patient had been on paclitaxel chemotherapy since her diagnosis, with a regimen that included paclitaxel 145 mg IV and dexamethasone 10 mg IV. Her home medications are summarized in Table 1. The patient denied any alcohol use, and there was no documented history of alcohol consumption in the medical record. Moreover, there was no documented history of diabetes, dyslipidemia, or metabolic syndrome, and no lipid panel was available prior to the initiation of paclitaxel therapy.

**Table 1:** Home medications mg: milligrams; OD: once a day

Indication	Medication	Dose
High Blood Pressure	Amlodipine 2.5 mg tablet	2.5 mg oral once a day (OD)
	Metoprolol Succinate 100 mg tab extended release	100 mg oral once a day
	Triamterene 37.5 mg-Hydrochlorthiazide 25 mg capsule	1 capsule oral once a day
Pain or Fever	Ibuprofen 600 mg tablet	1 tablet oral every 6 hours as needed, a total of 20 tablets
	Oxycodone-Acetaminophen 10 mg-325 mg tablet	1 tablet oral every 6 hours as needed, a total of 15 tablets
Nausea and Vomiting	Ondansetrone 4 mg disintegrating tablet	4 mg oral every 4 hours as needed, a total of 15 tablets

The patient had a few prior ED visits for nausea, vomiting, and abdominal pain; however, during these prior visits, no formal diagnosis of acute pancreatitis was established, and she was discharged after symptomatic management. She subsequently presented to the ED on 12th February 2023, one week after receiving her chemotherapy, with a complaint of sudden-onset severe epigastric abdominal pain radiating to the back. The pain was associated with nausea and vomiting episodes, which caused shortness of breath. Physical examination showed epigastric tenderness. Upon further evaluation, laboratory tests revealed elevated levels of serum lipase, triglycerides, aspartate aminotransferase (AST), and alanine aminotransferase (ALT). The lipid panel, serum calcium, and liver function test (LFT) results are summarized in Table 2.

**Table 2:** Pertinent investigations and laboratory results AST: Aspartate Aminotransferase; ALT: Alanine Aminotransferase; LDL: Low-Density Lipoprotein; HDL: High-Density Lipoprotein; LFT: Liver Function Test “—” indicates a laboratory value not measured during that visit. Baseline pre-chemotherapy lipid values were unavailable in the medical record. LDL cannot be accurately calculated when triglycerides are >400 mg/dL. Time points are aligned to clinical course: ED visits, admission, and hospital days.

Date/Investigation	Serum Lipase (23-300 U/L)	Serum Triglycerides (<150 mg/dl)	Serum Cholesterol (<200 mg/dl)	LDL Cholesterol <100 mg/dl	Non-HDL Cholesterol (<130 mg/dl)	HDL Cholesterol (>= 60 mg/dl is desirable)	AST (3-45U/L)	ALT (0-35U/L)
12/11/22: ED Visit	669	—	—	—	—	—	—	—
01/16/23: ED Visit	—	3209	234	Unable to calculate	205	29	—	—
02/12/23: Admission Day 1	3359	—	—	—	—	—	53	53
02/14/23: Hospital Day 3	404	>1575	282	Unable to Calculate	260	22	28	29
02/15/23: Hospital Day 4	—	1294	320	Unable to Calculate	299	21	25	25
02/16/23: Hospital Day 5	—	1071	—	—	—	—	24	25
02/18/23: Hospital Day 7	264	689	—	—	—	—	—	—
Trend	↓	↓	↑	—	↑	↓	—	—

Although baseline lipid values prior to initiation of paclitaxel therapy were not available, serial laboratory results from the patient’s emergency department visits in December 2022 and January 2023 demonstrated marked abnormalities, including elevated lipase (669 U/L on 12/11/22) and severe hypertriglyceridemia (3,209 mg/dL on 01/16/23). These abnormalities preceded the February 2023 admission during which acute pancreatitis was diagnosed, suggesting progressive lipid derangement during ongoing paclitaxel therapy. A summary timeline of the lipase and triglyceride trends is shown in Table 3.

**Table 3:** Timeline of triglyceride and lipase levels prior to and during pancreatitis diagnosis ED: Emergency department

Date	Lipase (U/L)	Triglycerides (mg/dl)	Clinical Context
12/11/22	669	Not Measured	Initial ED Visit
01/16/23	Not Measured	3209	Severe Hypertriglyceridemia
02/12/23	3359	Not Measured	Recurrence of Abdominal Pain which triggered ED visit resulting in patient admission from 2/12-2/18
02/14/23	404	>1575	CT confirmed Pancreatitis
02/15/23	Not Measured	1294	Improving
02/16/23	Not Measured	1071	Improving
02/18/23	264	689	Clinical Recovery

**Figure 1:**
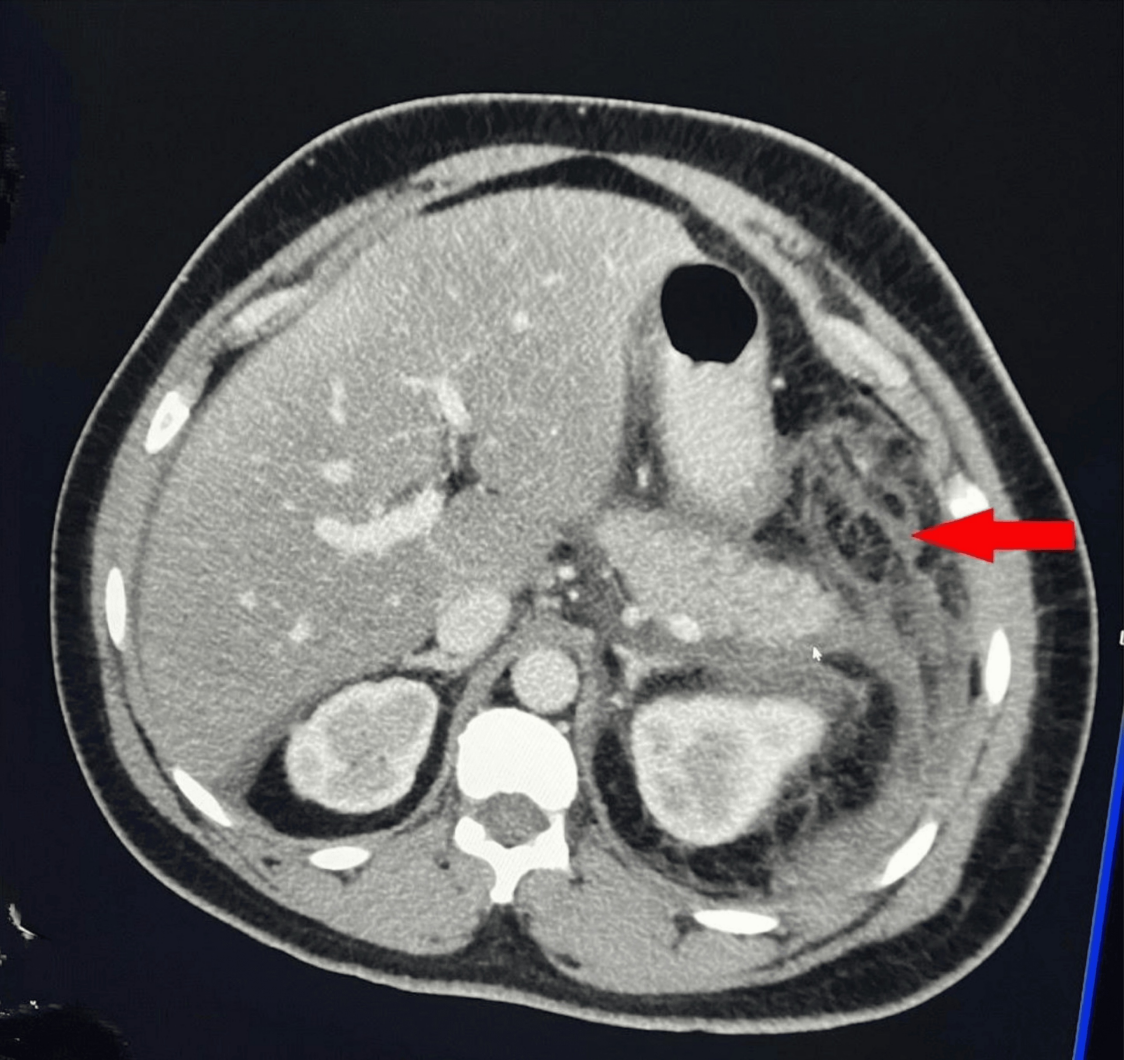
Axial view of the CT scan demonstrates extensive pancreatic inflammation and peripancreatic fat stranding consistent with acute pancreatitis (indicated by the red arrow) CT: Computed tomography

**Figure 2:**
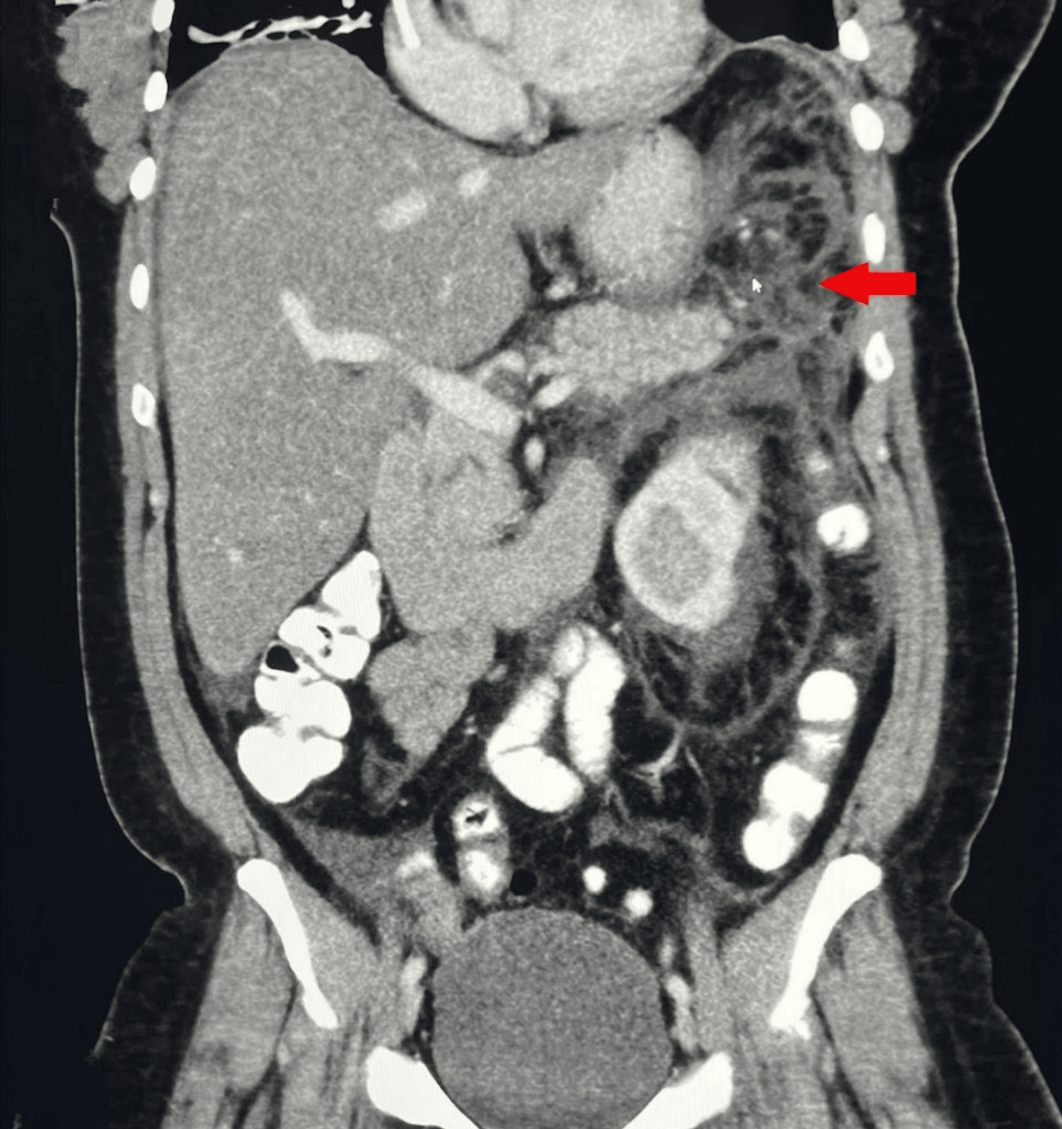
The coronal view of the CT scan reveals significant pancreatic inflammation consistent with acute pancreatitis (indicated by the red arrow) CT: Computed tomography

An abdominal ultrasound (USG) was performed on admission, which showed poor visualization of the pancreas and two 1 cm gallbladder stones. However, there was no evidence of pericholecystic fluid collection, gallbladder wall thickening, or intrahepatic biliary duct dilation. A CT scan of the abdomen and pelvis (Figures 1, 2) performed the following day revealed extensive infiltration around the pancreas, consistent with significant pancreatitis. It also showed inflammation of the left colon and splenic flexure, secondary inflammation of the stomach, duodenum, and loops of jejunum in the left upper quadrant, along with mild to moderate ascites in the abdomen and pelvis. The diagnosis of acute pancreatitis was made using the Atlanta criteria, which requires the presence of at least two of the following three findings: abdominal pain suggestive of acute pancreatitis, serum amylase and/or lipase levels elevated to at least three times the normal level, and characteristic findings on imaging. Since the patient did not have any history of alcohol consumption, alcohol-induced pancreatitis was effectively excluded as a potential etiology. 

The patient was treated with analgesics, proton pump inhibitors, and intravenous hydration. She was also started on gemfibrozil 600 mg twice daily for her hypertriglyceridemia. Ezetimibe (10 mg once daily), which primarily lowers LDL cholesterol and has a minimal effect on triglycerides, was also initiated during hospitalization for broader lipid control rather than for the acute management of hypertriglyceridemia. Over the course of her hospitalization, her pain resolved, and her serum triglyceride levels decreased from 1575 mg/dL to 1071 mg/dL, reaching 698 mg/dL on the last day of her admission. Similarly, her serum lipase levels decreased from 3359 IU/L to 264 IU/L. No further imaging or lab evaluations were conducted due to her clinical improvement, and she was scheduled for follow-up in the outpatient clinic. Paclitaxel was discontinued at the time of diagnosis of acute pancreatitis due to concern for a drug-induced etiology and the potential risk of rechallenge. The patient was also referred back to her oncology team for reassessment of her breast cancer treatment regimen.

We suspected her pancreatitis to be chemotherapy-induced, likely related to her paclitaxel-based regimen. Our suspicion was supported by evidence from case reports indicating that paclitaxel can cause hypertriglyceridemia and dyslipidemia, which may precipitate acute pancreatitis.

## Discussion

Paclitaxel, a chemotherapy drug widely used to treat various cancers, has been associated with several side effects, including severe hypertriglyceridemia and acute pancreatitis. In fact, paclitaxel has been classified under Class 3b in the revised evidence-based classification for drug-induced acute pancreatitis. This classification indicates that while high-quality case reports exist, they primarily rely on rechallenge data without consistent latency, highlighting the moderate quality of evidence linking paclitaxel to acute pancreatitis [7]. Although paclitaxel-induced pancreatitis is rare, our case contributes to the growing body of literature supporting the need for vigilant monitoring of patients undergoing chemotherapy to identify and manage this potential complication.

While the precise mechanism by which paclitaxel induces hypertriglyceridemia remains unclear, it is thought to involve disruptions in lipid metabolism. Severe hypertriglyceridemia can, in turn, lead to acute pancreatitis, a potentially life-threatening condition characterized by inflammation of the pancreas. Koneshamoorthy et al. (2022) suggested that the excipient Cremophor EL (CrEL), used in paclitaxel formulations, plays a pivotal role in inducing hypertriglyceridemia [8]. CrEL disrupts lipid metabolism by upregulating angiopoietin-like 4 (ANGPTL4), a protein that inhibits lipoprotein lipase, reducing triglyceride clearance [8]. This mechanism explains the significant rise in triglyceride levels observed in paclitaxel-treated patients. Combined with dexamethasone premedication, which exacerbates lipid abnormalities, this emphasizes the importance of close monitoring of lipid profiles in high-risk individuals receiving paclitaxel [8].

In this report, we present the case of a 48-year-old woman with a history of breast cancer who developed acute pancreatitis following paclitaxel chemotherapy. Laboratory tests revealed significantly elevated serum triglyceride and lipase levels, and imaging studies confirmed the diagnosis of acute pancreatitis according to the Atlanta criteria. Upon reviewing the literature, we found that paclitaxel-induced acute pancreatitis is a rare occurrence. A comprehensive review identified only a few case reports, noting variability in the onset and severity of symptoms [9]. 

In our case, the patient had been receiving paclitaxel for several months before the diagnosis of acute pancreatitis, which could suggest a delayed temporal relationship. However, triglyceride and lipase levels were not monitored routinely during her earlier chemotherapy cycles, and the available laboratory data from December 2022 and January 2023 already demonstrated markedly elevated lipase and severe hypertriglyceridemia. This pattern indicates that lipid derangements were likely evolving over time and only became clinically apparent during the February 2023 admission. Thus, rather than a sudden onset after a long latency, the course appears consistent with gradually progressive, treatment-associated hypertriglyceridemia culminating in pancreatitis.

Although the patient had an elevated BMI, she had no documented history of diabetes, chronic dyslipidemia, or metabolic syndrome, and no pretreatment lipid profile was available. Moreover, while gallstones were noted on abdominal ultrasound, gallstone-related pancreatitis was considered less likely due to the absence of biliary ductal dilation, gallbladder wall thickening, or pericholecystic fluid on imaging, as well as the lack of a cholestatic liver enzyme pattern. The patient also denied alcohol use. Therefore, while some baseline metabolic risk cannot be excluded, the progressive hypertriglyceridemia observed during ongoing paclitaxel therapy suggests a drug-induced or drug-unmasked mechanism.

A key aspect of this case is the significant hypertriglyceridemia, which likely triggered acute pancreatitis. While hypertriglyceridemia is a recognized cause of acute pancreatitis, its association with paclitaxel is seldom documented. Several studies underscore the importance of recognizing hypertriglyceridemia as a potential complication of chemotherapy, and early detection and management are crucial to prevent severe outcomes [6].

A similar pattern of drug-induced hypertriglyceridemia leading to acute pancreatitis has been observed with tamoxifen therapy. Hussain et al. (2024) conducted a systematic review identifying 17 cases of tamoxifen-induced hypertriglyceridemia resulting in pancreatitis with varying severity [10]. The study highlights the importance of lipid monitoring during cancer therapy, particularly in patients with underlying risk factors such as hypertension or diabetes [10]. This reinforces the need for vigilant lipid monitoring in paclitaxel-treated patients, as seen in our case, to prevent severe complications [10].

In a published review of reported cases, the onset of pancreatitis occurred after one to five cycles of paclitaxel, with symptom onset ranging from hours to several weeks following administration [9]. Most reported cases demonstrated mild to moderate disease severity with resolution following conservative management and paclitaxel discontinuation, although rare cases of necrotizing pancreatitis have been described [9]. Similar to our patient, prior cases typically excluded gallstone disease and alcohol use as primary etiologies, strengthening the association with paclitaxel exposure. Our patient likewise developed severe biochemical pancreatitis following prolonged paclitaxel exposure with rapid improvement after drug discontinuation, aligning closely with previously reported patterns.

This case underscores the critical importance of monitoring lipid levels in patients receiving paclitaxel chemotherapy. Early identification and treatment of severe hypertriglyceridemia are essential to prevent progression to acute pancreatitis. In this instance, the patient responded well to conservative treatment, including fibrates and a cholesterol absorption inhibitor, resulting in a significant reduction in triglyceride levels and resolution of symptoms.

Longer-term oncologic and metabolic outcomes following discharge could not be assessed, as subsequent cancer care occurred outside the period of direct clinical follow-up available to the treating team.

## Conclusions

This case highlights a rare but clinically significant association between paclitaxel therapy, severe hypertriglyceridemia, and acute pancreatitis. While the temporal onset following paclitaxel exposure, the marked triglyceride elevation, and the patient’s improvement with lipid-lowering therapy make paclitaxel a strong causal consideration, absolute certainty cannot be established given the absence of baseline lipid values, the presence of gallstones, and the lack of rechallenge. Paclitaxel has been categorized as a Badalov Class IIIb agent for drug-induced pancreatitis, based on limited case report level evidence without consistent latency or rechallenge data, but with biologically plausible mechanisms. Nevertheless, this case reinforces the importance of monitoring triglyceride levels in patients receiving paclitaxel-based chemotherapy, particularly those with metabolic risk factors, as early detection and intervention may prevent severe or life-threatening complications. These observations contribute to the growing body of literature describing paclitaxel-associated metabolic disturbances and highlight the need for further research to clarify the underlying mechanisms, better define epidemiology, and refine risk stratification.
